# Supplementary material for: How social distancing, mobility, and preventive policies affect COVID-19 outcomes: Big data-driven evidence from the District of Columbia-Maryland-Virginia (DMV) megaregion
Source: PLoS One. 2022 Feb 17;17(2):e0263820. doi: 10.1371/journal.pone.0263820 (PMC8853552; doi:10.1371/journal.pone.0263820)
Supplement: Attachment — Submitted filename: Response to Reviewers.docx [file pone.0263820.s001.docx]

**Response to Reviewer Comments**

**Title:** How social distancing, mobility, and preventive policies affect COVID-19 outcomes: Big data-driven evidence from the District of Columbia-Maryland-Virginia (DMV) megaregion

**Manuscript ID:** PONE-D-21-15442

Dear Dr. Li, Academic Editor of PLOS ONE,

We would like to thank you for reviewing our manuscript and the opportunity to submit a revised manuscript to PLOS ONE. Below, please find our responses to each point raised by the academic editor and reviewer(s).

We hope these responses will address the concerns raised and provide sufficient clarification to make our manuscript eligible for publication in PLOS ONE. Thank you again for your time and consideration.

***Part 1: Responses to Comments Regarding Journal Requirement***

When submitting your revision, we need you to address these additional requirements:

**Comment 1.** Please ensure that your manuscript meets PLOS ONE’s style requirements, including those for file naming. The PLOS ONE style templates can be found at https://journals.plos.org/plosone/s/file?id=wjVg/PLOSOne_formatting_sample_main_body.pdf and https://journals.plos.org/plosone/s/file?id=ba62/PLOSOne_formatting_sample_title_authors_affiliations.pdf

**Response to Comment 1:**

We have double checked our manuscript to make sure that it meets PLOS ONE’s style requirements including those mentioned in the “PLOS ONE MANUSCRIPT BODY FORMATTING GUIDELINES” and “PLOS ONE TITLE, AUTHOR, AFFILIATIONS FORMATTING GUIDELINES” templates.

**Comment 2.** We note that you have stated that you will provide repository information for your data at acceptance. Should your manuscript be accepted for publication, we will hold it until you provide the relevant accession numbers or DOIs necessary to access your data. If you wish to make changes to your Data Availability statement, please describe these changes in your cover letter and we will update your Data Availability statement to reflect the information you provide.

**Response to Comment 2:**

We have provided this information on GitHub via the following link:

<https://github.com/umdcxiong/social_distancing_DMV>

**Comment 3.** PLOS requires an ORCID iD for the corresponding author in Editorial Manager on papers submitted after December 6th, 2016. Please ensure that you have an ORCID iD and that it is validated in Editorial Manager. To do this, go to ‘Update my Information’ (in the upper left-hand corner of the main menu), and click on the Fetch/Validate link next to the ORCID field. This will take you to the ORCID site and allow you to create a new iD or authenticate a pre-existing iD in Editorial Manager. Please see the following video for instructions on linking an ORCID iD to your Editorial Manager account: https://www.youtube.com/watch?v=_xcclfuvtxQ.

**Response to Comment 3:**

The corresponding author has followed the mentioned steps and has validated his ORCID iD in Editorial Manager as requested.

**Comment 4.** We note that Figure 3 & 4 in your submission contain [map/satellite] images which may be copyrighted. All PLOS content is published under the Creative Commons Attribution License (CC BY 4.0), which means that the manuscript, images, and Supporting Information files will be freely available online, and any third party is permitted to access, download, copy, distribute, and use these materials in any way, even commercially, with proper attribution. For these reasons, we cannot publish previously copyrighted maps or satellite images created using proprietary data, such as Google software (Google Maps, Street View, and Earth). For more information, see our copyright guidelines: http://journals.plos.org/plosone/s/licenses-and-copyright.

We require you to either (1) present written permission from the copyright holder to publish these figures specifically under the CC BY 4.0 license, or (2) remove the figures from your submission:

a. You may seek permission from the original copyright holder of Figures 3 & 4 to publish the content specifically under the CC BY 4.0 license.

We recommend that you contact the original copyright holder with the Content Permission Form (http://journals.plos.org/plosone/s/file?id=7c09/content-permission-form.pdf) and the following text:

“I request permission for the open-access journal PLOS ONE to publish XXX under the Creative Commons Attribution License (CCAL) CC BY 4.0 (http://creativecommons.org/licenses/by/4.0/). Please be aware that this license allows unrestricted use and distribution, even commercially, by third parties. Please reply and provide explicit written permission to publish XXX under a CC BY license and complete the attached form.”

Please upload the completed Content Permission Form or other proof of granted permissions as an "Other" file with your submission.

In the figure caption of the copyrighted figure, please include the following text: “Reprinted from [ref] under a CC BY license, with permission from [name of publisher], original copyright [original copyright year].”

b. If you are unable to obtain permission from the original copyright holder to publish these figures under the CC BY 4.0 license or if the copyright holder’s requirements are incompatible with the CC BY 4.0 license, please either i) remove the figure or ii) supply a replacement figure that complies with the CC BY 4.0 license. Please check copyright information on all replacement figures and update the figure caption with source information. If applicable, please specify in the figure caption text when a figure is similar but not identical to the original image and is therefore for illustrative purposes only.

The following resources for replacing copyrighted map figures may be helpful:

USGS National Map Viewer (public domain): http://viewer.nationalmap.gov/viewer/

The Gateway to Astronaut Photography of Earth (public domain): http://eol.jsc.nasa.gov/sseop/clickmap/

Maps at the CIA (public domain): https://www.cia.gov/library/publications/the-world-factbook/index.html and https://www.cia.gov/library/publications/cia-maps-publications/index.html

NASA Earth Observatory (public domain): http://earthobservatory.nasa.gov/

Landsat: http://landsat.visibleearth.nasa.gov/

USGS EROS (Earth Resources Observatory and Science (EROS) Center) (public domain): http://eros.usgs.gov/#

Natural Earth (public domain): http://www.naturalearthdata.com/

**Response to Comment 4:**

Kindly note that Figures 3 and 4 in our manuscript are not copyrighted images/maps. These figures have been created by the authors using the ArcGIS Software, which is a tool for processing and analyzing geospatial data. University of Maryland and our research team at the Maryland Transportation Institute has active licenses for ArcGIS Software and we have created and published many maps/figures using this tool in the past. We certify that Figures 3 and 4 in our manuscript are not previously copyrighted maps or satellite images as they were created by us, the authors of this manuscript.

Please also note that no proprietary data, such as Google software (Google Maps, Street View and Earth or similar sources) were used in creating Figures 3 and 4. To create these figures, we used the following data: a) “TIGER/Line Shapefiles” (2020 county-level data), which are openly available to the public through the United States Census Bureau web site at <https://www.census.gov/geographies/mapping-files/time-series/geo/tiger-line-file.2021.html>; and b) the COVID-19 Data Repository by the Center for Systems Science and Engineering (CSSE) at Johns Hopkins University, which are also publicly accessible from the CSSE’s GitHub at: <https://github.com/CSSEGISandData/COVID-19>.

Based on these reasons, we—as the creators of Figures 3 and 4 in the manuscript—are the copyright holder for them; thus, publishing these presents no violation against the Creative Commons Attribution License (CC BY 4.0).

**Comment 5.** Please review your reference list to ensure that it is complete and correct. If you have cited papers that have been retracted, please include the rationale for doing so in the manuscript text, or remove these references and replace them with relevant current references. Any changes to the reference list should be mentioned in the rebuttal letter that accompanies your revised manuscript. If you need to cite a retracted article, indicate the article’s retracted status in the References list and also include a citation and full reference for the retraction notice.

**Response to Comment 5:**

We have double-checked all of the references in our reference list to make sure that is correct and updated. We do not have any retracted references; however, we had to make changes to a few of the references due to updates to the reference URLs/titles/dates.

More specifically, we updated our reference numbers 12, 25, 33, 34, 38, and 41—all of which are CDC web site references—due to the minor updates to the CDC web pages for these references. The blue-highlighted portions of the text below indicate where revisions to these references are made based on updates to the relevant web page. Basically, these revisions include an update to the title of the articles and/or the dates where these updates were posted on the corresponding CDC web page.

Please also note that we checked the updated content of all of these web pages to make sure that the relevant arguments made in the manuscript are still present in the updated references, and that was true for all the updated references.

***Old reference 12.*** The Centers for Disease Control and Prevention (CDC). Social Distancing. 2020 November 17. Available from: https://www.cdc.gov/coronavirus/2019-ncov/prevent-getting-sick/social-distancing.html.

***New reference 12.*** The Centers for Disease Control and Prevention (CDC). How to Protect Yourself & Others. 2021 November 29. Available from: https://www.cdc.gov/coronavirus/2019-ncov/prevent-getting-sick/social-distancing.html.

***Old reference 25.*** The Centers for Disease Control and Prevention (CDC). People at Increased Risk. 2021 Jan 4. Available from: https://www.cdc.gov/coronavirus/2019-ncov/need-extra-precautions/people-at-increased-risk.html?CDC_AA_refVal=https%3A%2F%2Fwww.cdc.gov%2Fcoronavirus%2F2019-ncov%2Fneed-extra-precautions%2Fpeople-at-higher-risk.html.

***New reference 25.*** The Centers for Disease Control and Prevention (CDC). COVID-19 Information for Specific Groups of People. At Increased Risk for Severe Illness. 2021 Apr 20. Available from: https://www.cdc.gov/coronavirus/2019-ncov/need-extra-precautions/index.html.

***Old reference 33***. The Centers for Disease Control and Prevention (CDC). When to Quarantine. 2020 Dec 10. Available from: https://www.cdc.gov/coronavirus/2019-ncov/if-you-are-sick/quarantine.html.

***New reference 33.*** The Centers for Disease Control and Prevention (CDC). Quarantine and Isolation. 2021 Oct 19. Available from: https://www.cdc.gov/coronavirus/2019-ncov/your-health/quarantine-isolation.html?CDC_AA_refVal=https%3A%2F%2Fwww.cdc.gov%2Fcoronavirus%2F2019-ncov%2Fif-you-are-sick%2Fquarantine.html.

***Old reference 34.*** The Centers for Disease Control and Prevention (CDC). Symptoms of Coronavirus. 2020 Dec 22. Available from: https://www.cdc.gov/coronavirus/2019-ncov/symptoms-testing/symptoms.html.

***New reference 34.*** The Centers for Disease Control and Prevention (CDC). Symptoms of Coronavirus. 2021 Feb 22. Available from: https://www.cdc.gov/coronavirus/2019-ncov/symptoms-testing/symptoms.html.

***Old reference 38.*** The Centers for Disease Control and Prevention (CDC). Running Essential Errands. 2020 Dec 31. Available from: https://www.cdc.gov/coronavirus/2019-ncov/daily-life-coping/essential-goods-services.html.

***New reference 38.*** The Centers for Disease Control and Prevention (CDC). Activities, Gatherings & Holidays. 2021 Aug 16. Available from: https://www.cdc.gov/coronavirus/2019-ncov/daily-life-coping/index.html?CDC_AA_refVal=https%3A%2F%2Fwww.cdc.gov%2Fcoronavirus%2F2019-ncov%2Fdaily-life-coping%2Fgoing-out.html.

***Old reference 41.*** The Centers for Disease Control and Prevention (CDC). Considerations for Wearing Masks. 2020 Dec 18. Available from: https://www.cdc.gov/coronavirus/2019-ncov/prevent-getting-sick/cloth-face-cover-guidance.html.

***New reference 41.*** The Centers for Disease Control and Prevention (CDC). Guidelines for Wearing Masks. 2021 Apr 19. Available from: https://www.cdc.gov/coronavirus/2019-ncov/prevent-getting-sick/cloth-face-cover-guidance.html.

***Part 2: Responses to Reviewer Comments***

**Reviewer #1:** COVID-19 has infected more than 188 million people and results in more than 4 million deaths since its first report. The pandemic leads to enormous damage to human health and the global economy. Social distancing, wearing face coverings and restricting travels have proven to be effective in slowing the spread of infections. The effects of human mobility restrictions on the spread of COVID-19 have also been widely investigated around the world. This study provides a link between human mobility changes and COVID-19 infections within a megaregion that contains the U.S. capital city. Several concerns need to be addressed.

Dear Reviewer,

We greatly appreciate your time and efforts on reviewing our manuscript. Your helpful comments will definitely improve this manuscript and for that, we thank you.

By providing detailed responses, we have tried to address each of your concerns to the best of our ability. Please see below for point-by-point responses to your comments.

**Comment 1.** How many mobile devices were included in this study and how to exclude the possible duplicated calculations as they are anonymized devices.

**Response to Comment 1:**

The MTI mobility data drew evidence from multiple mobile device data sources. This enables us to tap into a biggest and representative mobile device sample. Before integrating data and summarizing the total sample size, we have implemented a rigorous device deduplication based on data-driven imputation of home locations and most frequently visited locations. Devices that have the same imputed home location and shared the same top-five visited geohash zones will be recognized as duplicated samples and only one sample will be retained in the final integrated dataset. After the dedup and integration step, our final dataset observed over 80% of population of the DMV area. This is over 12 million devices observed on a monthly basis. We have added this sentence to the paper to clarify:

“After proper deduplication process, the average sampling rates for the three states are above 80%, indicating over 12 million devices were observed in our data pool.”

**Comment 2.** is there a specific standard for the “severe penalties” for violating stay-at-home orders which are linked to lower COVID-19 infection?

**Response to Comment 2:**

Due to various states having their own regulation and laws with respect to managing COVID-19, uniform and standard penalties against violating stay-at-home orders have not been in place. Therefore, the enforcement severity levels (i.e., penalties) imposed by the local (i.e., state) governments within the study area were defined in this study based on three levels of penalty severity (please see Table 1 in the manuscript). These three levels included:

Level 1 = confinement in jail for ≤ 12 months and a fine of ≤ $5,000, or both (enforcement in Maryland);

Level 2 = confinement in jail for ≤ 12 months and a fine of ≤ $2,500, or both (enforcement in Virginia);

Level 3 = confinement in jail for ≤ 90 days and a fine of ≤ $5,000, or both (enforcement in D.C.).

For modeling purposes, we considered Level 1 to be the most severe penalty and Level 3 to be the least severe penalty in our analysis. The results indicate that as the enforcement becomes less severe, the daily rates of COVID-19 infections and deaths within the county increase. This implies that stricter enforcement policies and heavier penalties for noncompliance with stay-at-home orders can help in decreasing the COVID-19 infections and deaths.

**Comment** **3.** Both the mobilities within communities and long-distance travel play an important role

in the spreading of the pandemic. Did the authors distinguish the two kinds of mobilities in this study?

**Response to Comment 3:**

We included various travel-related variables in our analysis including a variable representing the total number of external trips by infected persons from out of state/county (i.e., the *imported cases* variable), which is a proxy for long-distance travel.

We also included a variable representing the average number of all trips made per person per day (i.e., the *trips/person* variable) and a variable representing the average person-miles traveled on all modes per person per day (i.e., the *miles/person* variable). While the latter two variables do not distinguish between long- and short-distance trips, they certainly account for both types of mobility (long- and short-distance travel) through including all trips made by all modes of travel (i.e., walking, bicycling, automobile, transit, etc.) in the analysis.

**Comment 4.** Line 216. The wording is not accurate. there have been a lot of studies revealed the effects of human mobility on the virus spread based on large scale dataset, for example, Ying Zhou et al, 2020, Lancet Digital Health; Moritz U. G. Kraemer et al, 2020, Science; Pierre Nouvellet et al, 2021, Nature Communications.

**Response to Comment 4:**

To address this concern, we have done the following:

- We deleted the sentence in the manuscript on Line 216 (the sentence “*One shortcoming of previous research is the lack of a large-scale dataset that describes human mobility trends during the pandemic.”)*; and
- We revised the subsequent wording to: “*The present study aims to contribute to the empirical knowledge by using big mobile-device data providing information on human mobility changes during the COVID-19 pandemic within a megaregion that contains the U.S. capital city.”*

We hope that our responses to your comments address the concerns raised. Thank you again for your time and helpful feedback on our manuscript.
